# Supplementary material for: Clinical effectiveness and components of Home-pulmonary rehabilitation for people with chronic respiratory diseases: a systematic review protocol
Source: BMJ Open. 2021 Oct 12;11(10):e050362. doi: 10.1136/bmjopen-2021-050362 (PMC8513265; doi:10.1136/bmjopen-2021-050362)
Supplement: Supplementary data [file bmjopen-2021-050362supp001.pdf]

Appendix 1

Search of PubMed on 12th October 2020	Search results
((((home-based rehabilitation) OR (home-based rehabilitation program) OR (home-based training) OR (home-based program) OR (home-based pulmonary rehabilitation) OR (home-based pulmonary rehabilitation program) OR (home-based exercise training) OR (home exercise) OR (home-based exercise program) OR (home-based exercise) OR (community-based rehabilitation) OR (home care services) OR (telerehabilitation) OR (tele-rehabilitation) OR (telehealth) OR (tele-health) OR (teleconsultation) OR (tele-consultation) OR (real-time videoconferencing) OR (videoconferencing) OR (telerehabilitation[MeSH Terms]) OR (home care services[MeSH Terms]))) <br>AND ((lung disease) OR (pulmonary disease) OR (respiratory disease) OR (chronic respiratory disease) OR (chronic obstructive pulmonary disease) OR (COPD) OR (chronic obstructive airway disease) OR (chronic obstructive lung disease) OR (chronic airflow obstruction) OR (post tb) OR (post-tuberculosis) OR (interstitial lung disease) OR (idiopathic pulmonary fibrosis) OR (idiopathic interstitial pneumonia) OR (asthma) OR (occupational lung disease) OR (pulmonary hypertension) OR (lung transplant) OR (chronic bronchitis) OR (emphysema) OR (lung diseases, interstitial[MeSH Terms]) OR (bronchiectasis[MeSH Terms]) OR (idiopathic interstitial pneumonia[MeSH Terms]) OR (idiopathic pulmonary fibrosis[MeSH Terms]) OR (pulmonary disease, chronic obstructive[MeSH Terms]) OR (lung transplantation[MeSH Terms]))) <br>AND ((pulmonary rehabilitation) OR (cardiopulmonary rehabilitation) OR (respiratory therapy) OR (respiratory muscle training) OR (breathing exercise) OR (pulmonary exercise) OR (pre-habilitation) OR (breathing exercises[MeSH Terms]) OR (respiratory therapy[MeSH Terms])) AND (1990:2020[pdat])	4058
