# Supplementary material for: Clinical effectiveness and components of Home-pulmonary rehabilitation for people with chronic respiratory diseases: a systematic review protocol
Source: BMJ Open. 2021 Oct 12;11(10):e050362. doi: 10.1136/bmjopen-2021-050362 (PMC8513265; doi:10.1136/bmjopen-2021-050362)
Supplement: Author's manuscript [file bmjopen-2021-050362.draft_revisions.pdf]

BMJ Open

BMJ Open is committed to open peer review. As part of this commitment we make the peer review history of every article we publish publicly available.

When an article is published we post the peer reviewers' comments and the authors' responses online. We also post the versions of the paper that were used during peer review. These are the versions that the peer review comments apply to.

The versions of the paper that follow are the versions that were submitted during the peer review process. They are not the versions of record or the final published versions. They should not be cited or distributed as the published version of this manuscript.

BMJ Open is an open access journal and the full, final, typeset and author-corrected version of record of the manuscript is available on our site with no access controls, subscription charges or pay-per-view fees (http://bmjopen.bmj.com).

If you have any questions on BMJ Open's open peer review process please email info.bmjopen@bmj.com

BMJ Open

Clinical effectiveness, and components of home-pulmonary rehabilitation for people with chronic respiratory diseases: A systematic review protocol

Journal:	*BMJ Open*
Manuscript ID	bmjopen-2021-050362
Article Type:	Protocol
Date Submitted by the Author:	20-Feb-2021
Complete List of Authors:	Uzzaman, Md. Nazim; The University of Edinburgh<br>Chan, Soo Chin; University of Malaya,<br>Ranita, Hisham; University of Malaya<br>Engkasan, Julia; University of Malaya<br>Agarwal, Dhiraj ; KEM Hospital Pune Research Centre<br>Habib, GM Monsur; The University of Edinburgh<br>Hanafi, Nik Sherina; University of Malaya<br>Jackson, Tracy; The University of Edinburgh<br>Jebaraj, Paul; Christian Medical College Vellore,<br>Khoo, Ee Ming; University of Malaya<br>Liew, Su May; University of Malaya<br>Mirza, Fatim; Universiti Teknologi MARA, Centre for Physiotherapy Studies<br>Pinnock, Hilary; The University of Edinburgh<br>Rabinovich, Roberto; The University of Edinburgh Centre for Inflammation Research,
Keywords:	Chronic airways disease < THORACIC MEDICINE, RESPIRATORY MEDICINE (see Thoracic Medicine), Rehabilitation medicine < INTERNAL MEDICINE

SCHOLARONE™
Manuscripts

I, the Submitting Author has the right to grant and does grant on behalf of all authors of the Work (as defined in the below author licence), an exclusive licence and/or a non-exclusive licence for contributions from authors who are: i) UK Crown employees; ii) where BMJ has agreed a CC-BY licence shall apply, and/or iii) in accordance with the terms applicable for US Federal Government officers or employees acting as part of their official duties; on a worldwide, perpetual, irrevocable, royalty-free basis to BMJ Publishing Group Ltd ("BMJ") its licensees and where the relevant Journal is co-owned by BMJ to the co-owners of the Journal, to publish the Work in this journal and any other BMJ products and to exploit all rights, as set out in our licence.

The Submitting Author accepts and understands that any supply made under these terms is made by BMJ to the Submitting Author unless you are acting as an employee on behalf of your employer or a postgraduate student of an affiliated institution which is paying any applicable article publishing charge ("APC") for Open Access articles. Where the Submitting Author wishes to make the Work available on an Open Access basis (and intends to pay the relevant APC), the terms of reuse of such Open Access shall be governed by a Creative Commons licence – details of these licences and which Creative Commons licence will apply to this Work are set out in our licence referred to above.

Other than as permitted in any relevant BMJ Author's Self Archiving Policies, I confirm this Work has not been accepted for publication elsewhere, is not being considered for publication elsewhere and does not duplicate material already published. I confirm all authors consent to publication of this Work and authorise the granting of this licence.

For peer review only

Clinical effectiveness, and components of home-pulmonary rehabilitation for people with chronic respiratory diseases: A systematic review protocol

Authors:

1. Md. Nazim Uzzaman, NIHR Global Health Research Unit on Respiratory Health (RESPIRE), University of Edinburgh, Edinburgh, UK
   Email: nazim0700@gmail.com
2. Soo Chin Chan, Faculty of Medicine, University of Malaya, Kuala Lumpur, Malaysia
   Email: scchan@ummc.edu.my
3. Ranita Hisham Shunmugam, Library, University of Malaya, Kuala Lumpur, Malaysia
   Email: ranita@um.edu.my
4. Julia Patrick Engkasan, Faculty of Medicine, University of Malaya, Kuala Lumpur, Malaysia
   Email: julia@ummc.edu.my
5. Dhiraj Agarwal, KEM Hospital and Research Centre, Pune, India
   Email: dhiraj.agarwal@kemhrcvadu.org
6. GM Monsur Habib, NIHR Global Health Research Unit on Respiratory Health (RESPIRE), University of Edinburgh, Edinburgh, UK
   Email: gmmhabib@gmail.com
7. Nik Sherina Hanafi, Faculty of Medicine, University of Malaya, Kuala Lumpur, Malaysia
   Email: sherina@ummc.edu.my
8. Tracy Jackson, NIHR Global Health Research Unit on Respiratory Health (RESPIRE), The University of Edinburgh, Edinburgh, UK
   Email: tracy.jackson@ed.ac.uk
9. Paul Jebaraj, Christian Medical College, Vellore, India
   Email: pauljebaraj15@gmail.com
10. Ee Ming Khoo, Faculty of Medicine, University of Malaya, Kuala Lumpur, Malaysia Email: khooem@um.edu.my
11. Su May Liew, Faculty of Medicine, University of Malaya, Kuala Lumpur, Malaysia Email: smliew@ummc.edu.my
12. Fatim Tahirah Mirza, Faculty of Health Sciences, Universiti Teknologi MARA, Selangor, Malaysia
    Email: fatim_mirza@uitm.edu.my
13. Hilary Pinnock, NIHR Global Health Research Unit on Respiratory Health (RESPIRE), The University of Edinburgh, Edinburgh, UK
    Email: hilary.pinnock@ed.ac.uk
14. Roberto A. Rabinovich, University of Edinburgh, Edinburgh, UK
    Email: roberto.rabinovich@ed.ac.uk

Correspondence:

Dr Roberto A. Rabinovich

Pulmonologist, Respiratory Department, Royal Infirmary Edinburgh, and

ELEGI/Colt laboratory, Centre for Inflammation Research, QMRI,

University of Edinburgh

Tel: +44 0131 2429198

E-mail: roberto.rabinovich@ed.ac.uk

Word count: 1735

For peer review only - http://bmjopen.bmj.com/site/about/guidelines.xhtml

ABSTRACT

Introduction: Chronic respiratory diseases (CRDs) are common and disabling conditions that can result in social isolation and economic hardship for patients and their families. Pulmonary rehabilitation (PR) improves functional exercise capacity and health-related quality of life (HRQoL) but practical barriers to attending centre-based sessions or the need for infection control limits accessibility. Home-PR offers a potential solution that may improve access. We aim to systematically review the clinical effectiveness, completion rates and components of Home-PR for people with CRDs compared to Centre-PR or Usual care.

Methods and analysis: We will search PubMed, CINAHL, Cochrane, EMBASE, PeDRO, and PsycInfo from January 1990 to date using a PICOS search strategy (Population: adults with CRDs); Intervention: Home-PR; Comparator: Centre-PR/Usual care; Outcomes: functional exercise capacity and HRQoL; Setting: any setting). Six reviewers working in pairswill independently screen articles for eligibility and extract data from those fulfilling the inclusion criteria. We will use the Cochrane risk-of bias tool and GRADE approach to rate the quality of evidence. We will perform meta-analysis or narrative synthesis as appropriate to answer our three research questions: 1) What is the effectiveness of Home-PR compared to Centre-PR or Usual care?2) What components are used in effective Home-PR studies? and, 3) What is the completion rate of Home-PR compared to Centre-PR?

Ethics and dissemination: Research ethics approval is not required since the study will review only published data. The findings will be disseminated through publication in a peer-reviewed journal, and presentation in conferences.

PROSPERO registration number: CRD42020220137

Key words: Chronic Respiratory Diseases, Chronic Obstructive Pulmonary Disease, Pulmonary Rehabilitation, Home-Pulmonary Rehabilitation, Systematic Review.

For peer review only

ARTICLE SUMMARY

Strengths and limitations of this study

- A systematic review of the effectiveness, completion rates and components of Home-PR for chronic respiratory diseases (CRDs) is needed to inform patients and providers especially when health care accessibility is restricted by geography, demography or during pandemics.
- The review methods are in accordance to Cochrane methodology and preferred reporting items for systematic reviews and meta-analyses (PRISMA) publishing guidelines.
- Issues like heterogeneity, poor reporting of published trials may affect confidence in results although we expect to provide robust evidence supporting the successful implementation of Home-PR services for people with CRDs.
- The multi-disciplinary, multinational research team will enable a nuanced interpretation of the findings.

For peer review only

INTRODUCTION

Chronic respiratory diseases (CRDs) including chronic obstructive pulmonary disease (COPD), remodelled asthma, pulmonary impairment after tuberculosis (PIAT), interstitial lung disease (ILD), bronchiectasis and cystic fibrosis (CF), among others, affect an estimated 545 million people globally.[1] Around four million people die prematurely from CRDs each year across the world,[2] and COPD, tuberculosis and asthma are all within the top 30 conditions responsible for high rates of disability-adjusted life-years.[3] CRDs, in particular COPD, are associated with breathlessness, fatigue, and muscle dysfunction which contribute to reduced physical activity levels and functional exercise capacity.[4] This functional impairment is related to reduced health-related quality-of-life (HRQoL), increased adverse events and mortality independent of the level of airway obstruction.[5, 6]

Pulmonary rehabilitation (PR) is an essential component of CRD care[7] that improves functional exercise capacity, HRQoL and reduces the burden of chronic respiratory symptoms.[8, 9] It is defined as a comprehensive, multidisciplinary, and multifaceted intervention based on a thorough patient assessment, followed by individually-tailored therapies that are designed to improve the physical and psychological conditions of people with CRDs and to support the long-term adherence to health-enhancing behaviours.[10, 11] The components of PR include exercise training, education, nutritional support, smoking cessation, lifestyle modification, and self-management, among others. PR is indicated for patients who continue to experience symptoms despite optimising pharmacological treatment.[12-14]

Despite proven effectiveness,[15, 16] PR is under-utilised. The reasons for poor attendance and completion rates are multifactorial and commonly identified barriers include: low referral rate; inconvenient timing of the programmes necessitating time off work; geographical distance to PR centres which can be made worse in some countries by poor transportinfrastructure.[17-21] Whilst pertinent even in high income countries[22-24] many of these barriers are exacerbated in low- and middle-income countries (LMICs) where there is a lack of structured PR facilities especially in rural communities.[25, 26]

Typically PR is provided in centres (Centre-PR),[27] but globally different models are tailored to the local context such as Community-PR,[28] and Home-PR with telephone-mentoring,[29] or tele-rehabilitation programmes.[30] The ongoing coronavirus disease (COVID-19) pandemic has added strain to PR services by increasing the population for whom PR is indicated and adding barriers to the delivery of the treatment due to cross-infection issues. There is, therefore, an increased interest

For peer review only

in Home-PR[31] as a strategy to overcome these barriers. A Cochrane review of 65 studies (3822patients) has established the effectiveness of standard Centre-PR programmes in COPD[15] with some evidence in bronchiectasis[11] and ILD.[32] A systematic review in 2016 suggested that Home/Community-PR (the review did not distinguish these two approaches) could be as effective as Centre-PR for people with COPD.[33] However, these programmes were heterogeneous and did not identify components with greater impact on positive patient outcomes. We therefore aim to systematically review the literature to assess the effectiveness, completion rates and components used in effective Home-PR for people with CRDs.

OBJECTIVES

In people with CRDs, we will:

1. Assess the clinical effectiveness of Home-PR (see Table 1 for definition) compared to Centre-PR or Usual care at improving health outcomes (i.e. exercise capacity [primary outcome], HRQoL [primary outcome], dyspnoea, muscle fatigue, exacerbations and hospitalisations for CRD)
2. Describe the components of Home-PR that are associated with successful interventions (e.g., intensity of exercise, duration of the programme, education and/or other non-exercise components, frequency of supervision, information/resources, involvement of family members)
3. Compare the completion rate (defined as participating in at least 75% of PR sessions) of Home-PR with Centre-PR

METHODS AND ANALYSIS

We will follow Cochrane methodology,[34] and use PRISMA guidelines[35] to report our review findings. The review is registered with PROSPERO [ID: CRD42020220137], any changes to the published record will be reported.

Search strategy

We will develop a search strategy, and identify records through searching the following databases: PubMed, CINAHL, Cochrane, EMBASE, PeDRO, and PsycInfo (appendix 1). The strategy will search for ‘Chronic Respiratory Disease’ AND ‘Pulmonary Rehabilitation’ AND ‘Home-PR’ from 1990, when global COPD guidelines first recommended PR.[36] We will check reference lists and conduct forward citation on included studies and on Cochrane reviews of PR.[15] We will not impose any language restriction, and will arrange for translation to English to enable selection and data extraction.[37]

For peer review only

Selection process

We will select studies that compare Home-PR for people with CRDs with Centre-PR and/or Usual care (see definitions and details of our PICOS criteria in Table 1). Following training on 100 randomly selected records, six reviewers (MNU, TJ, JPE, FT, DA, PJ working in pairs) will duplicate screen titles and abstracts and identify potentially eligible studies. Disagreements will be resolved by discussion with the review team (HP, RR, SML, MH, NSH and SC) as necessary. After retrieval of the full text of potentially eligible studies, six reviewers (MNU, TJ, JPE, FT, DA, PJ) will independently screen the studies against the selection criteria. Disagreements will be resolved by discussion within the team to arbitrate and determine rules for operationalising the inclusion/exclusion criteria. Anythingthat remains unclear, will be clarified by contacting the authors; if this fails, the study will be listed as 'potentially relevant study'. All processes will be reported in a PRISMA flow diagram[35], and excluded full-text papers will be tabulated with reasons for exclusion.

Outcome measurement

Our primary outcomes will be HRQoL and functional exercise capacity. We are interested in pre- and post-assessment or if an immediate post is not provided, the nearest figure to that. See Table 1 for details, and description of secondary outcomes.

Data management and extraction

We will develop a customised data extraction form based on Cochrane EPOC guidance.[38] This form will be piloted by all the researchers to standardised use and revised to endure that it captures all relevant information including the PICOS criteria, definitions used and outcome measurements. Data extraction will be carried out by six reviewers working in pairs (MNU, TJ, JPE, FT, DA, PJ). General information such as date of extraction, name of reviewer, article title, trial eligibility including type of study, participants, methods, number of participants in each group, reference of trials, intervention group, co-interventions, serious adverse events, description of funding, ethical approval will be extracted from included full-text papers. We will contact authors for any missing data.

Risk of bias assessment

Methodological quality of all included articles will be assessed independently by reviewers(MNU,TJ, JPE, FT, DA, PJ working in pairs) using the 'Cochrane Risk of Bias'tool.[34] Discrepancies will be resolved by discussion with the team. We will assess the papers for selection, performance, detection, attrition, reporting and other sources of bias, and assess the overall risk of bias. We will record and tabulate a summary of the assessment with the overall judgement.

For peer review only - http://bmjopen.bmj.com/site/about/guidelines.xhtml

Heterogeneity and reporting bias

We will assess and investigate reasons for any heterogeneity using the I^2 statistic[39] and create a funnel plot to test for publication bias[40] unless we have fewer than 10 trials.

Data analysis

Objective 1: We plan to undertake meta-analysis for the primary outcomes and some secondary outcomes (e.g., HRQoL, dyspnoea, muscle fatigue, exacerbations, hospitalisations), comparing Home-PR with a) Centre-PR or b) Usual care. Heterogeneous outcomes for which a meta-analysis is inappropriate will be synthesised narratively.

Objective 2: The components of Home-PR will be described and a matrix compiled to identify any associations with successful interventions.

Objective 3: If appropriate, we will undertake a meta-analysis of completion rates comparing Home-PR and Centre-PR.

Sub-group analyses

Depending on the papers included, we will perform subgroup analyses. Subgroups may include high/low- and middle-income countries, CRD diagnosis (e.g., COPD, ILD, bronchiectasis), severity as defined in internationally recognised guidelines, intensity of intervention (number of weeks, sessions per week, workload), and arrangements of supervision and level of supervision of the PR programme.

Interpretation of findings

We will use the GRADE approach[41] to assess the quality of evidence and strength of recommendations for the primary outcomes and the important secondary outcomes (listed in Table 1).

Patients and public involvement

Patients are involved in the with the RESPIRE programme of work on pulmonary rehabilitation. They have endorsed the importance of Home-PR for improving accessibility for patients, and will be involved in interpreting the findings and the implications for intervention development and the overall programme of work. We are grateful to the RESPIRE patient colleagues who have offered their advice on the pulmonary rehabilitation programme work.

For peer review only

DISCUSSION

Home-PR has particular resonance at the time of developing this protocol because of the COVID-19 pandemic which has resulted in Centre-PR services being halted. More generally, there is an interest in offering Home-PR as a strategy to overcome the practical barriers of time and distance and increase accessibility of PR services especially in LMICs. There are, however, concerns that the relative lack of supervision and the loss of peer group support may reduce effectiveness. Hence a review on the effectiveness of Home-PR and its components is timely to inform patients, professionals and healthcare service providers considering Home-PR options.

ETHICS AND DISSEMINATION

This systematic review protocol will use publicly available data without direct involvement of human participants. Therefore, approval from an ethics committee is not essential. We will present our review findings at national and international scientific meetings and conferences, and publish in a peer-reviewed journal. In addition, we will use innovative dissemination strategies including virtual seminars and social media.

AUTHOR CONTRIBUTIONS

RR and HP led the team who all contributed to the development of the protocol. MNU drafted the first version of the manuscript with support from SCC and DA, which was revised with contributions from all the authors. All authors have critically reviewed and approved the final manuscript.

DATA STATEMENT

Not applicable. This is a protocol. All results will be made available in the final publication.

FUNDING

This research was commissioned by the UK National Institute for Health Research (NIHR) Global Health Research Unit on Respiratory Health (RESPIRE): 16/136/109, using UK Aid from the UK Government. The views expressed in this publication are those of the author(s) and not necessarily those of the NIHR or the UK Department of Health and Social Care. The RESPIRE collaboration comprises the UK Grant holders, Partners and research teams as listed on the RESPIRE website (www.ed.ac.uk/usher/respire), including Harish Nair. TJ is part-funded by the NIHR RESPIRE. MH has a NIHR RESPIRE PhD studentship. MNU, DA, SML hold NIHR RESPIRE Fellowships. HP, RR, EMK, SCC, JPE, NSH, SML are co-investigators of NIHR RESPIRE-funded PR feasibility studies in their respective Centres. RESPIRE is funded by the National Institute of Health Research using Official Development Assistance (ODA) funding.

COMPETING INTERESTS

MH owns a Pulmonary Rehabilitation clinic in Bangladesh. All other authors declare no competing interests.

For peer review only

REFERENCES

1. Soriano JB, Kendrick PJ, Paulson KR, *et al.* Prevalence and attributable health burden of chronic respiratory diseases, 1990–2017: a systematic analysis for the Global Burden of Disease Study 2017. *Lancet Respir Med* 2020; **8**: 585-596.
2. Organization WH. The Global Impact of Respiratory Disease. *World Health Organization: Sheffield* 2017.
3. Hay SI, Abajobir AA, Abate KH, *et al.* Global, regional, and national disability-adjusted life-years (DALYs) for 333 diseases and injuries and healthy life expectancy (HALE) for 195 countries and territories, 1990–2016: a systematic analysis for the Global Burden of Disease Study 2016. *Lancet* 2017; **390**: 1260-1344.
4. Garcia-Aymerich J, FéLEZ MA, Escarrabill J, *et al.* Physical activity and its determinants in severe chronic obstructive pulmonary disease. *Med Sci Sports Exerc* 2004; **36**: 1667-1673.
5. Brien SB, Lewith GT and Thomas M. Patient coping strategies in COPD across disease severity and quality of life: a qualitative study. *NPJ Prim Care Respir Med* 2016; **26**: 1-7.
6. Hesselink AE, Van Der Windt D, Penninx B, *et al.* What predicts change in pulmonary function and quality of life in asthma or COPD? *J Asthma* 2006; **43**: 513-519.
7. Troosters T, Blondeel A, Rodrigues FM, *et al.* Strategies to Increase Physical Activity in Chronic Respiratory Diseases. *Clin Chest Med* 2019; **40**: 397-404. 2019/05/13. DOI: 10.1016/j.ccm.2019.02.017.
8. Spruit MA, Singh SJ, Garvey C, *et al.* An official American Thoracic Society/European Respiratory Society statement: key concepts and advances in pulmonary rehabilitation. *Am J Respir Crit Care Med* 2013; **188**: e13-e64.
9. Singh D, Agusti A, Anzueto A, *et al.* Global strategy for the diagnosis, management, and prevention of chronic obstructive lung disease: the GOLD science committee report 2019. *Eur Respir J* 2019; **53**.
10. Rochester CL, Vogiatzis I, Holland AE, *et al.* An official American Thoracic Society/European Respiratory Society policy statement: enhancing implementation, use, and delivery of pulmonary rehabilitation. *Am J Respir Crit Care Med* 2015; **192**: 1373-1386.
11. Bradley JM, Moran F and Greenstone M. Physical training for bronchiectasis. *Cochrane Database Syst Rev* 2002.
12. *Global Initiative for Chronic Obstractive Lung Disease. Pocket guide to COPD diagnosis, management and prevention.* 2019.
13. Bolton CE, Bevan-Smith EF, Blakey JD, *et al.* British Thoracic Society guideline on pulmonary rehabilitation in adults. *Thorax* 2013; **68 Suppl 2**: ii1-30. 2013/08/02. DOI: 10.1136/thoraxjnl-2013-203808.
14. Rochester CL, Vogiatzis I, Holland AE, *et al.* An Official American Thoracic Society/European Respiratory Society Policy Statement: Enhancing Implementation, Use, and Delivery of Pulmonary Rehabilitation. *Am J Respir Crit Care Med* 2015; **192**: 1373-1386. 2015/12/02. DOI: 10.1164/rccm.201510-1966ST.
15. McCarthy B, Casey D, Devane D, *et al.* Pulmonary rehabilitation for chronic obstructive pulmonary disease. *Cochrane Database Syst Rev* 2015.
16. Puhan MA, Gimeno-Santos E, Cates CJ, *et al.* Pulmonary rehabilitation following exacerbations of chronic obstructive pulmonary disease. *Cochrane Database Syst Rev* 2016.
17. Alsubaiei ME, Cafarella PA, Frith PA, *et al.* Barriers for setting up a pulmonary rehabilitation program in the Eastern Province of Saudi Arabia. *Ann Thorac Med* 2016; **11**: 121.
18. Cox NS, Oliveira CC, Lahham A, *et al.* Pulmonary rehabilitation referral and participation are commonly influenced by environment, knowledge, and beliefs about consequences: a systematic review using the Theoretical Domains Framework. *J Physiother* 2017; **63**: 84-93.
19. Keating A, Lee A and Holland AE. What prevents people with chronic obstructive pulmonary disease from attending pulmonary rehabilitation? A systematic review. *Chron Respir Dis* 2011; **8**: 89-99.

For peer review only

20. Romain D, Bernady A, Etchamendy E, *et al.* Costs of hospitalisation for exacerbations of COPD in patients receiving domiciliary rehabilitation. *Rev Mal Respir* 2011; **28**: 864.
21. Trish E, Xu J and Joyce G. Medicare beneficiaries face growing out-of-pocket burden for specialty drugs while in catastrophic coverage phase. *Health Aff* 2016; **35**: 1564-1571.
22. Brooks D, Lacasse Y and Goldstein RS. Pulmonary rehabilitation programs in Canada: national survey. *Can Respir J* 1999; **6**: 55.
23. Wadell K, Ferreira TJ, Arne M, *et al.* Hospital-based pulmonary rehabilitation in patients with COPD in Sweden–a national survey. *Respir Med* 2013; **107**: 1195-1200.
24. Yohannes AM and Connolly MJ. Pulmonary rehabilitation programmes in the UK: a national representative survey. *Clin Rehabil* 2004; **18**: 444-449.
25. Desalu OO, Onyedum CC, Adeoti AO, *et al.* Guideline-based COPD management in a resource-limited setting—physicians' understanding, adherence and barriers: a cross-sectional survey of internal and family medicine hospital-based physicians in Nigeria. *Prim Care Respir J* 2013; **22**: 79-85.
26. Habib GM, Rabinovich R, Divgi K, *et al.* Systematic review of clinical effectiveness, components, and delivery of pulmonary rehabilitation in low-resource settings. *NPJ Prim Care Respir Med* 2020; **30**: 1-14.
27. Man WD, Puhan MA, Harrison SL, *et al.* Pulmonary rehabilitation and severe exacerbations of COPD: solution or white elephant? *ERJ Open Res* 2015; **1**.
28. Farias CC, Resqueti V, Dias FA, *et al.* Costs and benefits of pulmonary rehabilitation in chronic obstructive pulmonary disease: a randomized controlled trial. *Braz J Phys Ther* 2014; **18**: 165-173.
29. Bove DG, Overgaard D, Lomborg K, *et al.* Efficacy of a minimal home-based psychoeducative intervention versus usual care for managing anxiety and dyspnoea in patients with severe chronic obstructive pulmonary disease: a randomised controlled trial protocol. *BMJ Open* 2015; **5**.
30. Franke K-J, Domanski U, Schroeder M, *et al.* Telemonitoring of home exercise cycle training in patients with COPD. *Int J Chron Obstruct Pulmon Dis* 2016; **11**: 2821.
31. Yang F, Liu N, Hu J, *et al.* Pulmonary rehabilitation guidelines in the principle of 4S for patients infected with 2019 novel coronavirus (2019-nCoV). *Zhonghua Jie He He Hu Xi Za Zhi* 2020; **43**: 180-182.
32. Dowman L, Hill CJ and Holland AE. Pulmonary rehabilitation for interstitial lung disease. *Cochrane Database Syst Rev* 2014.
33. Neves LF, Reis MHd and Gonçalves TR. Home or community-based pulmonary rehabilitation for individuals with chronic obstructive pulmonary disease: a systematic review and meta-analysis. *Cad Saude Publica* 2016; **32**: e00085915.
34. HigginsJPT G. Cochrane handbook for systematic reviews of interventions version 5.1. 0 [updated March 2011]. *The Cochrane Collaboration* 2011; 20011.
35. Liberati A, Altman DG, Tetzlaff J, *et al.* The PRISMA statement for reporting systematic reviews and meta-analyses of studies that evaluate health care interventions: explanation and elaboration. *J Clin Epidemiol* 2009; **62**: e1-e34.
36. Casaburi R. A brief history of pulmonary rehabilitation. *Respir Care* 2008; **53**: 1185-1189.
37. Fung IC. Citation of non-English peer review publications–some Chinese examples. *Emerg Themes Epidemiol* 2008; **5**: 12.
38. EPC A. The Cochrane effective practice and organisation of care group. 2002.
39. Higgins JP, Thompson SG, Deeks JJ, *et al.* Measuring inconsistency in meta-analyses. *BMJ* 2003; **327**: 557-560. 2003/09/06. DOI: 10.1136/bmj.327.7414.557.
40. Egger M, Smith GD, Schneider M, *et al.* Bias in meta-analysis detected by a simple, graphical test. *BMJ* 1997; **315**: 629-634. DOI: 10.1136/bmj.315.7109.629.
41. Schünemann H, Brożek J, Guyatt G, *et al.* GRADE handbook for grading quality of evidence and strength of recommendations. Updated October 2013. The GRADE Working Group, 2013. *Available from guidelinedevelopment org/handbook* 2013.

For peer review only

Table 1. PICOS table for the search strategy

PICOS	Description, inclusion	Exclusion criteria	Operational rules
Population	• Adults with primary diagnosis of CRDs.<br>• Age> 18 years.<br>• Comorbidity will not be an exclusion criterion	• Pregnant women and paediatric population<br>• Rehabilitation provided to predominant condition is non-respiratory conditions<br>• Recovery from acute infections or injury (e.g., immediately post-COVID) until the condition has been stable for 6-months<br>• Conference abstract<br>• Lung cancer<br>• Pulmonary hypertension	PR delivered to people with chronic respiratory diseases (CRDs) such as chronic obstructive pulmonary disease (COPD), remodelled asthma, pulmonary impairment after tuberculosis (PIAT), bronchiectasis, interstitial lung disease (ILD), cystic fibrosis (CF), stable post-COVID (but excluding post-ICU rehabilitation) will be studied. We will also include PR delivered to people with more than one CRD, or undifferentiated chronic respiratory conditions.
Intervention	Home-Pulmonary Rehabilitation (PR) which comprises both exercise and at least one non-exercise component for a duration not lesser than 4 weeks.	Formal hospital or community medical centre-based programmes	**'Home-PR'-** the key criterion is that the sessions are undertaken by an individual by themselves (though a family member may be involved) and typically at home. The patient does not attend a Centre, is not directly supervised by a healthcare professional (though they may be monitored remotely for some or all of the session).<br>Exercise sessions typically include aerobic, endurance, resistance, and reconditioning exercises, though local resources and preferences may include other exercise modalities. Non-exercise components commonly include patient education, energy conservation training, smoking cessation, psychological support, self-management skill development or other recognised PR interventions along with optimisation of pharmacotherapy
Comparison	Either Population receiving 'Centre-PR' or receiving 'Usual care'.	No control group	**'Centre-PR'-** the key criterion is that the sessions are under direct healthcare professional's supervision. The 'Centre' can be in a hospital, community setting, or remote facility. Centre-based sessions are normally group-based (though it is recognised that this may be modified in the context of a pandemic). Telehealth services where patients attend a supervised satellite Centre would be considered as Centre-PR.<br><br>**'Usual care'**- is the standard care received by individual with CRD in the relevant healthcare system but excluding the exercise components of PR.

For peer review only

Outcomes	Consist of either one of the following outcome measures<br>• Health-Related Quality of Life (HRQoL)<br>• Functional exercise capacity<br>±<br>Additional outcome(s)<br>• Uptake of the service, completion rates<br>• Assessment of motivation/others intermediate outcome<br>• Activities of daily living<br>• Physical activity level<br>• Symptom control<br>• Psychological status<br>• Health care burden e.g., exacerbation rates, hospitalisation etc.<br>• Adverse effect	Not including HRQoL or any measurement of exercise capacity as outcome	Validated instruments will be considered:<br>• HRQoL: St Georges Respiratory Questionnaire (SGRQ), Chronic Respiratory Questionnaire (CRQ), EuroQol Five Dimension (EQ-5D)<br>• Functional exercise capacity: 6-Minute Walk Test (6MWT), Incremental Shuttle Walking Test (ISWT), Endurance Shuttle Walking Test (ESWT)<br>• Symptom control: Modified Medical Research Council (mMRC), Clinical COPD Questionnaire (CCQ), Borg scale<br>• Psychological status: Hospital Anxiety and Depression Scale (HADS), Patient Health Questionnaire-9 (PHQ-9), State-Trait Anxiety Inventory (STAI), Beck Inventory test
Setting	Any settings		Low or high resource settings irrespective of level of economies of the countries.
Study designs	Randomised controlled trials (RCTs); Clinical controlled trials	Cohort study, case series, case report	We will exclude studies that do not have a control group.
Language	No language restriction		

For peer review only

Appendix 1

Search of PubMed on 12th October 2020	Search results
((((home-based rehabilitation) OR (home-based rehabilitation program) OR (home-based training) OR (home-based program) OR (home-based pulmonary rehabilitation) OR (home-based pulmonary rehabilitation program) OR (home-based exercise training) OR (home exercise) OR (home-based exercise program) OR (home-based exercise) OR (community-based rehabilitation) OR (home care services) OR (telerehabilitation) OR (tele-rehabilitation) OR (telehealth) OR (tele-health) OR (teleconsultation) OR (tele-consultation) OR (real-time videoconferencing) OR (videoconferencing) OR (telerehabilitation[MeSH Terms]) OR (home care services[MeSH Terms])))<br><br>AND ((lung disease) OR (pulmonary disease) OR (respiratory disease) OR (chronic respiratory disease) OR (chronic obstructive pulmonary disease) OR (COPD) OR (chronic obstructive airway disease) OR (chronic obstructive lung disease) OR (chronic airflow obstruction) OR (post tb) OR (post-tuberculosis) OR (interstitial lung disease) OR (idiopathic pulmonary fibrosis) OR (idiopathic interstitial pneumonia) OR (asthma) OR (occupational lung disease) OR (pulmonary hypertension) OR (lung transplant) OR (chronic bronchitis) OR (emphysema) OR (lung diseases, interstitial[MeSH Terms]) OR (bronchiectasis[MeSH Terms]) OR (idiopathic interstitial pneumonia[MeSH Terms]) OR (idiopathic pulmonary fibrosis[MeSH Terms]) OR (pulmonary disease, chronic obstructive[MeSH Terms]) OR (lung transplantation[MeSH Terms])))<br><br>AND ((pulmonary rehabilitation) OR (cardiopulmonary rehabilitation) OR (respiratory therapy) OR (respiratory muscle training) OR (breathing exercise) OR (pulmonary exercise) OR (pre-habilitation) OR (breathing exercises[MeSH Terms]) OR (respiratory therapy[MeSH Terms])) AND (1990:2020[pdat])	4058

For peer review only

Reporting checklist for protocol of a systematic review and meta analysis.

Based on the PRISMA-P guidelines.

Instructions to authors

Complete this checklist by entering the page numbers from your manuscript where readers will find each of the items listed below.

Your article may not currently address all the items on the checklist. Please modify your text to include the missing information. If you are certain that an item does not apply, please write "n/a" and provide a short explanation.

Upload your completed checklist as an extra file when you submit to a journal.

In your methods section, say that you used the PRISMA-Preporting guidelines, and cite them as:

Moher D, Shamseer L, Clarke M, Ghersi D, Liberati A, Petticrew M, Shekelle P, Stewart LA. Preferred Reporting Items for Systematic Review and Meta-Analysis Protocols (PRISMA-P) 2015 statement. Syst Rev. 2015;4(1):1.

		Reporting Item	Page Number
**Title**			
Identification	#1a	Identify the report as a protocol of a systematic review	2
Update	#1b	If the protocol is for an update of a previous systematic review, identify as such	n/a
**Registration**			
	#2	If registered, provide the name of the registry (such as PROSPERO) and registration number	PROSPERO CRD42020220137
**Authors**			

For peer review only

Contact	#3a	Provide name, institutional affiliation, e-mail address of all protocol authors; provide physical mailing address of corresponding author	1
Contribution	#3b	Describe contributions of protocol authors and identify the guarantor of the review	8
**Amendments**			
	#4	If the protocol represents an amendment of a previously completed or published protocol, identify as such and list changes; otherwise, state plan for documenting important protocol amendments	n/a
**Support**			
Sources	#5a	Indicate sources of financial or other support for the review	8
Sponsor	#5b	Provide name for the review funder and / or sponsor	8
Role of sponsor or funder	#5c	Describe roles of funder(s), sponsor(s), and / or institution(s), if any, in developing the protocol	8
**Introduction**			
Rationale	#6	Describe the rationale for the review in the context of what is already known	4
Objectives	#7	Provide an explicit statement of the question(s) the review will address with reference to participants, interventions, comparators, and outcomes (PICO)	5,11,12
**Methods**			
Eligibility criteria	#8	Specify the study characteristics (such as PICO, study design, setting, time frame) and report characteristics (such as years considered, language, publication status) to be used as criteria for eligibility for the review	11, 12
Information sources	#9	Describe all intended information sources (such as electronic databases, contact with study authors, trial registers or other grey literature sources) with planned dates of coverage	5

For peer review only

Search strategy	#10	Present draft of search strategy to be used for at least one electronic database, including planned limits, such that it could be repeated	5
Study records - data management	#11a	Describe the mechanism(s) that will be used to manage records and data throughout the review	
Study records - selection process	#11b	State the process that will be used for selecting studies (such as two independent reviewers) through each phase of the review (that is, screening, eligibility and inclusion in meta-analysis)	6
Study records - data collection process	#11c	Describe planned method of extracting data from reports (such as piloting forms, done independently, in duplicate), any processes for obtaining and confirming data from investigators	6
Data items	#12	List and define all variables for which data will be sought (such as PICO items, funding sources), any pre-planned data assumptions and simplifications	6, 11, 12
Outcomes and prioritization	#13	List and define all outcomes for which data will be sought, including prioritization of main and additional outcomes, with rationale	6
Risk of bias in individual studies	#14	Describe anticipated methods for assessing risk of bias of individual studies, including whether this will be done at the outcome or study level, or both; state how this information will be used in data synthesis	6
Data synthesis	#15a	Describe criteria under which study data will be quantitatively synthesised	7
Data synthesis	#15b	If data are appropriate for quantitative synthesis, describe planned summary measures, methods of handling data and methods of combining data from studies, including any planned exploration of consistency (such as I2, Kendall's τ)	7
Data synthesis	#15c	Describe any proposed additional analyses (such as sensitivity or subgroup analyses, meta-regression)	7
Data synthesis	#15d	If quantitative synthesis is not appropriate, describe the type of summary planned	7

For peer review only

Meta-bias(es)	#16	Specify any planned assessment of meta-bias(es) (such as publication bias across studies, selective reporting within studies)	6
Confidence in cumulative evidence	#17	Describe how the strength of the body of evidence will be assessed (such as GRADE)	7

None The PRISMA-P elaboration and explanation paper is distributed under the terms of the Creative Commons Attribution License CC-BY. This checklist can be completed online using https://www.goodreports.org/, a tool made by the EQUATOR Network in collaboration with Penelope.ai

For peer review only

BMJ Open

Clinical effectiveness, and components of home-pulmonary rehabilitation for people with chronic respiratory diseases: A systematic review protocol

Journal:	*BMJ Open*
Manuscript ID	bmjopen-2021-050362.R1
Article Type:	Protocol
Date Submitted by the Author:	04-Sep-2021
Complete List of Authors:	Uzzaman, Md. Nazim; The University of Edinburgh<br>Chan, Soo Chin; University of Malaya,<br>Ranita, Hisham; University of Malaya<br>Engkasan, Julia; University of Malaya<br>Agarwal, Dhiraj ; KEM Hospital Pune Research Centre<br>Habib, GM Monsur; The University of Edinburgh<br>Hanafi, Nik Sherina; University of Malaya<br>Jackson, Tracy; The University of Edinburgh<br>Jebaraj, Paul; Christian Medical College Vellore,<br>Khoo, Ee Ming; University of Malaya<br>Liew, Su May; University of Malaya<br>Mirza, Fatim; Universiti Teknologi MARA, Centre for Physiotherapy Studies<br>Pinnock, Hilary; The University of Edinburgh<br>Rabinovich, Roberto; The University of Edinburgh Centre for Inflammation Research,
**Primary Subject Heading**:	Respiratory medicine
Secondary Subject Heading:	Rehabilitation medicine
Keywords:	Chronic airways disease < THORACIC MEDICINE, RESPIRATORY MEDICINE (see Thoracic Medicine), Rehabilitation medicine < INTERNAL MEDICINE

SCHOLARONE™
Manuscripts

I, the Submitting Author has the right to grant and does grant on behalf of all authors of the Work (as defined in the below author licence), an exclusive licence and/or a non-exclusive licence for contributions from authors who are: i) UK Crown employees; ii) where BMJ has agreed a CC-BY licence shall apply, and/or iii) in accordance with the terms applicable for US Federal Government officers or employees acting as part of their official duties; on a worldwide, perpetual, irrevocable, royalty-free basis to BMJ Publishing Group Ltd ("BMJ") its licensees and where the relevant Journal is co-owned by BMJ to the co-owners of the Journal, to publish the Work in this journal and any other BMJ products and to exploit all rights, as set out in our licence.

The Submitting Author accepts and understands that any supply made under these terms is made by BMJ to the Submitting Author unless you are acting as an employee on behalf of your employer or a postgraduate student of an affiliated institution which is paying any applicable article publishing charge ("APC") for Open Access articles. Where the Submitting Author wishes to make the Work available on an Open Access basis (and intends to pay the relevant APC), the terms of reuse of such Open Access shall be governed by a Creative Commons licence – details of these licences and which Creative Commons licence will apply to this Work are set out in our licence referred to above.

Other than as permitted in any relevant BMJ Author's Self Archiving Policies, I confirm this Work has not been accepted for publication elsewhere, is not being considered for publication elsewhere and does not duplicate material already published. I confirm all authors consent to publication of this Work and authorise the granting of this licence.

For peer review only

Clinical effectiveness, and components of home-pulmonary rehabilitation for people with chronic respiratory diseases: A systematic review protocol

Authors:

1. Md. Nazim Uzzaman, NIHR Global Health Research Unit on Respiratory Health (RESPIRE), University of Edinburgh, Edinburgh, UK
   Email: nazim0700@gmail.com
2. Soo Chin Chan, Faculty of Medicine, University of Malaya, Kuala Lumpur, Malaysia
   Email: scchan@ummc.edu.my
3. Ranita Hisham Shunmugam, Library, University of Malaya, Kuala Lumpur, Malaysia
   Email: ranita@um.edu.my
4. Julia Patrick Engkasan, Faculty of Medicine, University of Malaya, Kuala Lumpur, Malaysia
   Email: julia@ummc.edu.my
5. Dhiraj Agarwal, KEM Hospital and Research Centre, Pune, India
   Email: dhiraj.agarwal@kemhrcvadu.org
6. GM Monsur Habib, NIHR Global Health Research Unit on Respiratory Health (RESPIRE), University of Edinburgh, Edinburgh, UK
   Email: gmmhabib@gmail.com
7. Nik Sherina Hanafi, Faculty of Medicine, University of Malaya, Kuala Lumpur, Malaysia
   Email: sherina@ummc.edu.my
8. Tracy Jackson, NIHR Global Health Research Unit on Respiratory Health (RESPIRE), The University of Edinburgh, Edinburgh, UK
   Email: tracy.jackson@ed.ac.uk
9. Paul Jebaraj, Christian Medical College, Vellore, India
   Email: pauljebaraj15@gmail.com
10. Ee Ming Khoo, Faculty of Medicine, University of Malaya, Kuala Lumpur, Malaysia Email: khooem@um.edu.my
11. Su May Liew, Faculty of Medicine, University of Malaya, Kuala Lumpur, Malaysia Email: smliew@ummc.edu.my
12. Fatim Tahirah Mirza, Faculty of Health Sciences, Universiti Teknologi MARA, Selangor, Malaysia
    Email: fatim_mirza@uitm.edu.my
13. Hilary Pinnock, NIHR Global Health Research Unit on Respiratory Health (RESPIRE), The University of Edinburgh, Edinburgh, UK
    Email: hilary.pinnock@ed.ac.uk
14. Roberto A. Rabinovich, University of Edinburgh, Edinburgh, UK
    Email: roberto.rabinovich@ed.ac.uk

Correspondence:

Dr Roberto A. Rabinovich

Pulmonologist, Respiratory Department, Royal Infirmary Edinburgh, and

ELEGI/Colt laboratory, Centre for Inflammation Research, QMRI,

University of Edinburgh

Tel: +44 0131 2429198

E-mail: roberto.rabinovich@ed.ac.uk

Word count: 2034

For peer review only

ABSTRACT

Introduction: Chronic respiratory diseases (CRDs) are common and disabling conditions that can result in social isolation and economic hardship for patients and their families. Pulmonary rehabilitation (PR) improves functional exercise capacity and health-related quality of life (HRQoL) but practical barriers to attending centre-based sessions or the need for infection control limits accessibility. Home-PR offers a potential solution that may improve access. We aim to systematically review the clinical effectiveness, completion rates and components of Home-PR for people with CRDs compared to Centre-PR or Usual care.

Methods and analysis: We will search PubMed, CINAHL, Cochrane, EMBASE, PeDRO, and PsycInfo from January 1990 to date using a PICOS search strategy (Population: adults with CRDs); Intervention: Home-PR; Comparator: Centre-PR/Usual care; Outcomes: functional exercise capacity and HRQoL; Setting: any setting). The strategy is to search for 'Chronic Respiratory Disease' AND 'Pulmonary Rehabilitation' AND 'Home-PR', and identify relevant randomised controlled trials and controlled clinical trials. Six reviewers working in pairs will independently screen articles for eligibility and extract data from those fulfilling the inclusion criteria. We will use the Cochrane risk-of bias tool and GRADE approach to rate the quality of evidence. We will perform meta-analysis or narrative synthesis as appropriate to answer our three research questions: 1) What is the effectiveness of Home-PR compared to Centre-PR or Usual care?2) What components are used in effective Home-PR studies? and, 3) What is the completion rate of Home-PR compared to Centre-PR?

Ethics and dissemination: Research ethics approval is not required since the study will review only published data. The findings will be disseminated through publication in a peer-reviewed journal, and presentation in conferences.

PROSPERO registration number: CRD42020220137

Key words: Chronic Respiratory Diseases, Chronic Obstructive Pulmonary Disease, Pulmonary Rehabilitation, Home-Pulmonary Rehabilitation, Systematic Review.

For peer review only

ARTICLE SUMMARY

Strengths and limitations of this study

- A systematic review of the effectiveness, completion rates and components of Home-PR for chronic respiratory diseases (CRDs) is needed to inform patients and providers especially when health care accessibility is restricted by geography, demography or during pandemics.
- The review methods are in accordance to Cochrane methodology and preferred reporting items for systematic reviews and meta-analyses (PRISMA) publishing guidelines.
- Issues like heterogeneity, poor reporting of published trials may affect confidence in results although we expect to provide robust evidence supporting the successful implementation of Home-PR services for people with CRDs.
- The multi-disciplinary, multinational research team will enable a nuanced interpretation of the findings.

For peer review only

INTRODUCTION

Chronic respiratory diseases (CRDs) including chronic obstructive pulmonary disease (COPD), remodelled asthma, pulmonary impairment after tuberculosis (PIAT), interstitial lung disease (ILD), bronchiectasis and cystic fibrosis (CF), among others, affect an estimated 545 million people globally.[1] Around four million people die prematurely from CRDs each year across the world,[2] and COPD, tuberculosis and asthma are all within the top 30 conditions responsible for high rates of disability-adjusted life-years.[3] CRDs, in particular COPD, are associated with breathlessness, fatigue, and muscle dysfunction which contribute to reduced physical activity levels and functional exercise capacity.[4] This functional impairment is related to reduced health-related quality-of-life (HRQoL), increased adverse events and mortality independent of the level of airway obstruction.[5,6]

Pulmonary rehabilitation (PR) is an essential component of CRD care[7] that improves functional exercise capacity, HRQoL and reduces the burden of chronic respiratory symptoms.[8,9] It is defined as a comprehensive, multidisciplinary, and multifaceted intervention based on a thorough patient assessment, followed by individually-tailored therapies that are designed to improve the physical and psychological conditions of people with CRDs and to support the long-term adherence to health-enhancing behaviours.[10,11] The components of PR include exercise training, education, nutritional support, smoking cessation, lifestyle modification, and self-management, among others. PR is indicated for patients who continue to experience symptoms despite optimising pharmacological treatment.[12,13]

Despite proven effectiveness,[11,14-15] PR is under-utilised. The reasons for poor attendance and completion rates are multifactorial and commonly identified barriers include: low referral rate; inconvenient timing of the programmes necessitating time off work; geographical distance to PR centres which can be made worse in some countries by poor transport infrastructure.[16-20] Whilst pertinent even in high income countries[21-23] many of these barriers are exacerbated in low- and middle-income countries (LMICs) where there is a lack of structured PR facilities especially in rural communities.[24,25]

Typically PR is provided in hospital centres (Centre-PR),[26] but globally different models are tailored to the local context such as Community-PR,[27] and Home-PR with telephone-mentoring,[28] or tele-rehabilitation programmes.[29] The ongoing coronavirus disease (COVID-19) pandemic has added strain to PR services by increasing the population for whom PR is indicated and adding barriers to the delivery of the treatment due to cross-infection issues. There is, therefore, an increased interest

For peer review only

in Home-PR[30] as a strategy to overcome these barriers. A Cochrane review of 65 studies (3822 patients) has established the effectiveness of standard Centre-PR programmes in COPD[14] with a sub-group analysis suggesting that PR delivered in a hospital centre may have a greater treatment effect than PR delivered in the community/home. Using the same definitions, three reviews (Wuytack et al,[31] Chen et al. 2020,[32] and Neves et al[33]) included studies comparing PR delivered in different settings and both concluded that home/community-PR could be as effective as Centre-PR for people with COPD. Combining home and community services, however, overlooks the distinction between a community-based group supervised in person by a healthcare professional and a programme delivered to an individual in their own home. These reviews are also limited by disease (COPD only), although there is evidence that PR is of benefit in bronchiectasis[11] and ILD.[34] Taito in a scoping review also included people recovering from COVID-19.[35]

A recently published Cochrane review assessed the effectiveness and safety of telerehabilitation for people with CRDs when compared to Centre-PR or no rehabilitation.[36] and concluded that primary or maintenance PR telerehabilitation achieved similar outcomes to Centre-PR. In this review, remote delivery of PR was defined by the use of telecommunications technology to deliver PR services to individuals or groups (either physical or virtual) in any location, including in the patient's home or at a healthcare centre. In contrast, in our review, the definition of Home-PR is that the sessions are undertaken by an individual by themselves (though a family member may be involved) and typically at home. Apart from baseline and post-PR assessments,[35] the patient does not attend a Centre (either a hospital Centre or a local 'satellite' Centre) and is not supervised face-to-face by a healthcare professional (though there may be remote communication from a healthcare professional for some or all of the session).

An additional distinction is that we defined Home-PR as comprising both exercise and at least one non-exercise component for a duration of not less than 4 weeks. This contrasts with other reviews [32,36,37] that included exercise training programmes (i.e. without the non-exercise component that is normally included in Centre-PR.[8]) These reviews did not seek to identify components with greater impact on positive patient outcomes. We therefore aim to systematically review the literature to assess the effectiveness, completion rates and components used in effective Home-PR for people with CRDs.

OBJECTIVES

In people with CRDs, we will:

For peer review only

1. Assess the clinical effectiveness of Home-PR (see Table 1 for definition) compared to Centre-PR or Usual care at improving health outcomes (i.e. exercise capacity [primary outcome], HRQoL [primary outcome], dyspnoea, muscle fatigue, exacerbations and hospitalisations for CRD)
2. Describe the components of Home-PR that are associated with successful interventions (e.g., intensity of exercise, duration of the programme, education and/or other non-exercise components, frequency of supervision, information/resources, involvement of family members)
3. Compare the completion rate (defined as participating in at least 70% of PR sessions) of Home-PR with Centre-PR

METHODS AND ANALYSIS

We will follow Cochrane methodology,[37] and use PRISMA guidelines[38] to report our review findings. The review is registered with PROSPERO [ID: CRD42020220137], any changes to the published record will be reported.

Search strategy

We will develop a search strategy, including disease-specific search terms, and identify records through searching the following databases: PubMed, CINAHL, Cochrane, EMBASE, PeDRO, and PsycInfo (Appendix 1). The strategy will search for 'Chronic Respiratory Disease' AND 'Pulmonary Rehabilitation' AND 'Home-PR' from 1990, when global COPD guidelines first recommended PR.[39] We will check reference lists and conduct forward citation on included studies and on Cochrane reviews of PR.[14,17] We will not impose any language restriction, and will arrange for translation to English to enable selection and data extraction.

Selection process

We will select studies that compare Home-PR for people with CRDs with Centre-PR and/or Usual care (see definitions and details of our PICOS criteria in Table 1). Following training on 100 randomly selected records, six reviewers working in pairs (MNU and TJ, JPE and FTM, DA and PJ) will duplicate screen titles and abstracts and identify potentially eligible studies. Disagreements will be resolved by discussion with the review team (HP, RR, SML, MH, NSH and SC) as necessary. After retrieval of the full text of potentially eligible studies, the six reviewers working in the same pairs will independently screen the studies against the selection criteria. Disagreements will be resolved by discussion within the team to arbitrate and determine rules for operationalising the inclusion/exclusion criteria. Anything that remains unclear, will be clarified by contacting the authors; if this fails, the study will

For peer review only

be listed as 'potentially relevant study'. All processes will be reported in a PRISMA flow diagram,[38] and excluded full-text papers will be tabulated with reasons for exclusion.

Outcome measurement

Our primary outcomes will be HRQoL and functional exercise capacity. We are interested in pre- and post-assessment or if an immediate post is not provided, the nearest figure to that. See Table 1 for details, and description of secondary outcomes.

Data management and extraction

We will develop a customised data extraction form based on Cochrane EPOC guidance.[40] This form will be piloted by all the researchers to standardised use and revised to endure that it captures all relevant information including the PICOS criteria, definitions used and outcome measurements. Data extraction will be carried out by six reviewers working in pairs (MNU, TJ, JPE, FTM, DA, PJ). General information such as date of extraction, name of reviewer, article title, trial eligibility including type of study, participants, methods, number of participants in each group, reference of trials, intervention group, co-interventions, serious adverse events, description of funding, ethical approval will be extracted from included full-text papers. We will contact authors for any missing data. If this is not possible and the missing data seem to introduce serious bias, we will perform sensitivity analysis of the impact of including such studies in the overall assessment of results.

Risk of bias assessment

Methodological quality of all included RCTs will be assessed independently by reviewers (MNU,TJ, JPE, FTM, DA, PJ working in pairs) using the 'Cochrane Risk of Bias'tool.[37] Discrepancies will be resolved by discussion with the team. We will assess the papers for selection, performance, detection, attrition, reporting and other sources of bias, and assess the overall risk of bias. We will record and tabulate a summary of the assessment with the overall judgement. To assess risk of bias of CCTs, we will use the 'Risk of Bias in Non-randomised Studies of Interventions' (ROBINS-I) tool.[41] We will include all studies in our primary analysis but take into account the risk of bias of the studies when considering the intervention effects. If there are sufficient studies we may undertake sensitivity analyses omitting studies at high risk of bias.

Heterogeneity and reporting bias

We will assess and investigate reasons for any heterogeneity using the I^2 statistic[42] and create a funnel plot to test for publication bias[43] unless we have fewer than 10 trials.

For peer review only

Data analysis

Objective 1: We plan to undertake meta-analysis for the primary outcomes and some secondary outcomes (e.g., HRQoL, dyspnoea, muscle fatigue, exacerbations, hospitalisations), comparing Home-PR firstly with Usual Care and then with Centre-PR. Heterogeneous outcomes for which a meta-analysis is inappropriate will be synthesised narratively. For homogenous data from RCTs, we will perform a pooled quantitative synthesis using an inverse variance method and a random-effects model in the meta-analysis. We will consider pooled mean differences if same outcome measurement tool is used in the included RCTs. However, if (as expected) outcome measurement tool varies among trials, we will consider standardised mean differences (SMDs). Our hypothesis is that Home-PR is non-inferior to Centre-PR, but a clinically meaningful non-inferiority margin cannot be inferred using SMDs. If sufficient studies use the same measure for functional exercise capacity or health-related quality of life, we will define the non-inferiority margin as the minimum clinically important difference. We will use Review Manager software (RevMan 2020, version 5.4.1) to perform meta-analysis.

Objective 2: The components of Home-PR will be described and a matrix compiled to identify any associations with successful interventions.

Objective 3: We will use a narrative approach to synthesise completion in Home-PR and Centre-PR groups. If sufficient studies report completion rates, we will consider a sub-group analysis based on the threshold of 70% completion.

Sub-group analyses

Depending on the papers included, we will perform subgroup analyses. Subgroups may include high/low- and middle-income countries, CRD diagnosis (e.g., COPD, ILD, bronchiectasis, stable post-COVID lung disease, mixed CRD), severity as defined in internationally recognised guidelines,[12] intensity of intervention (number of weeks, sessions per week, workload, completion rate), and arrangements for supervision of the PR programme.

Interpretation of findings

We will use the GRADE approach[44] to assess the quality of evidence and strength of recommendations for the primary outcomes and the important secondary outcomes (listed in Table 1).

For peer review only

Patient and public involvement

Patients who are involved in the RESPIRE programme of work on PR-have endorsed the importance of Home-PR for improving accessibility to rehabilitation. They will be involved in interpreting the findings and the implications for intervention development and the overall programme of work

DISCUSSION

Home-PR has particular resonance at the time of developing this protocol because of the COVID-19 pandemic which has resulted in Centre-PR services being halted. More generally, there is an interest in offering Home-PR as a strategy to overcome the practical barriers of time and distance and increase accessibility of PR services especially in LMICs. There are, however, concerns that the relative lack of supervision and the loss of peer group support may reduce effectiveness. Hence a review on the effectiveness of Home-PR and its components is timely to inform patients, professionals and healthcare service providers considering Home-PR options.

ETHICS AND DISSEMINATION

This systematic review protocol will use publicly available data without direct involvement of human participants. Therefore, approval from an ethics committee is not essential. We will present our review findings at national and international scientific meetings and conferences, and publish in a peer-reviewed journal. In addition, we will use innovative dissemination strategies including virtual seminars and social media.

For peer review only

AUTHOR CONTRIBUTIONS

RR and HP led the team (MNU, SCC, RHS, JPE, DA, GMMH, NSH, TJ, PJ, EMK, SML, FTM) who all contributed to the development of the protocol. MNU drafted the first version of the manuscript with support from SCC and DA, which was revised with contributions from all the authors. All authors have critically reviewed and approved the final manuscript.

DATA STATEMENT

Not applicable. This is a protocol. All results will be made available in the final publication.

ACKNOWLEDGEMENTS

We are grateful to the RESPIRE patient colleagues who have offered their advice on the pulmonary rehabilitation programme work.

FUNDING

This research was commissioned by the UK National Institute for Health Research (NIHR) Global Health Research Unit on Respiratory Health (RESPIRE): 16/136/109, using UK Aid from the UK Government. The views expressed in this publication are those of the author(s) and not necessarily those of the NIHR or the UK Department of Health and Social Care. The RESPIRE collaboration comprises the UK Grant holders, Partners and research teams as listed on the RESPIRE website (www.ed.ac.uk/usher/respire), including Harish Nair. TJ is part-funded by the NIHR RESPIRE. MH has a NIHR RESPIRE PhD studentship. MNU, DA, SML hold NIHR RESPIRE Fellowships. HP, RR, EMK, SCC, JPE, NSH, SML are co-investigators of NIHR RESPIRE-funded PR feasibility studies in their respective Centres. RESPIRE is funded by the National Institute of Health Research using Official Development Assistance (ODA) funding.

COMPETING INTERESTS

MH owns a Pulmonary Rehabilitation clinic in Bangladesh. All other authors declare no competing interests.

For peer review only

Table 1. PICOS table for the search strategy

PICOS	Description, inclusion	Exclusion criteria	Operational rules
Population	• Adults with primary diagnosis of CRDs.<br>• Age> 18 years.<br>• Comorbidity will not be an exclusion criterion	• Pregnant women and paediatric population<br>• Rehabilitation provided to predominant condition is non-respiratory conditions<br>• Recovery from acute infections or injury (e.g., immediately post-COVID) until the condition has been stable for 6-months<br>• Conference abstract<br>• Lung cancer<br>• Pulmonary hypertension	PR delivered to people with chronic respiratory diseases (CRDs) such as chronic obstructive pulmonary disease (COPD), remodelled asthma, pulmonary impairment after tuberculosis (PIAT), bronchiectasis, interstitial lung disease (ILD), cystic fibrosis (CF), stable post-COVID (but excluding post-ICU rehabilitation) will be studied. We will also include PR delivered to people with more than one CRD, or undifferentiated chronic respiratory conditions.
Intervention	Home-Pulmonary Rehabilitation (PR) which comprises both exercise and at least one non-exercise component for a duration not lesser than 4 weeks.	Formal hospital or community medical centre-based programmes	**'Home-PR'-** the key criterion is that the sessions are undertaken by an individual by themselves (though a family member may be involved) and typically at home. Apart from baseline and post-PR assessments,[35] the patient does not attend a Centre (either a hospital Centre or a local 'satellite' Centre) and is not supervised face-to-face by a healthcare professional (though there may be remote communication from a healthcare professional for some or all of the session)<br>Exercise sessions typically include aerobic, endurance, resistance, and reconditioning exercises, though local resources and preferences may include other exercise modalities. Non-exercise components commonly include patient education, energy conservation training, smoking cessation, psychological support, self-management skill development or other recognised PR interventions along with optimisation of pharmacotherapy
Comparison	Either Population receiving 'Centre-PR' or receiving 'Usual care'.	No control group	**'Centre-PR'-** the key criterion is that the sessions are under direct healthcare professional's supervision. The 'Centre' can be in a hospital, community setting, or remote facility. Centre-based sessions are normally group-based (though it is recognised that this may be modified in the context of a pandemic). Telehealth services where patients attend a supervised satellite Centre would be considered as Centre-PR.

For peer review only

			**'Usual care'**- is the standard care received by individual with CRD in the relevant healthcare system but excluding the exercise components of PR.
Outcomes	Consist of either one of the following outcome measures<br>• Health-Related Quality of Life (HRQoL)<br>• Functional exercise capacity<br>±<br>Additional outcome(s)<br>• Uptake of the service, completion rates<br>• Assessment of motivation/others intermediate outcome<br>• Activities of daily living<br>• Physical activity level<br>• Symptom control<br>• Psychological status<br>• Health care burden e.g., exacerbation rates, hospitalisation etc.<br>• Adverse effect	Not including HRQoL or any measurement of exercise capacity as outcome	Validated instruments will be considered:<br>• HRQoL: St Georges Respiratory Questionnaire (SGRQ), Chronic Respiratory Questionnaire (CRQ), EuroQol Five Dimension (EQ-5D)<br>• Functional exercise capacity: 6-Minute Walk Test (6MWT), Incremental Shuttle Walking Test (ISWT), Endurance Shuttle Walking Test (ESWT). We will also include step tests and sit-to-stand tests that are sometimes used in Home-PR assessments.[45]<br>• Symptom control: Modified Medical Research Council (mMRC), Clinical COPD Questionnaire (CCQ), Borg scale<br>• Psychological status: Hospital Anxiety and Depression Scale (HADS), Patient Health Questionnaire-9 (PHQ-9), State-Trait Anxiety Inventory (STAI), Beck Inventory test
Setting	Any settings		Low or high resource settings irrespective of level of economies of the countries.
Study designs	Randomised controlled trials (RCTs); Clinical controlled trials (CCTs)	Cohort study, case series, case report	We will exclude studies that do not have a control group. We will consider RCTs to answer all of the three research questions (i.e., 1. effectiveness, 2. components, and 3. completion rate of Home-PR.), and consider CCTs to answer research questions 2 and 3.
Language	No language restriction		

For peer review only

REFERENCES

1. Soriano JB, Kendrick PJ, Paulson KR*, et al.* Prevalence and attributable health burden of chronic respiratory diseases, 1990–2017: a systematic analysis for the Global Burden of Disease Study 2017. *Lancet Respir Med* 2020; **8:** 585-59
2. World Health Organization, The Global Impact of Respiratory Disease. *WHO*: Sheffield 2017
3. Hay SI, Abajobir AA, Abate KH*, et al.* Global, regional, and national disability-adjusted life-years (DALYs) for 333 diseases and injuries and healthy life expectancy (HALE) for 195 countries and territories, 1990–2016: a systematic analysis for the Global Burden of Disease Study 2016. *Lancet* 2017; **390:** 1260-134
4. Garcia-Aymerich J, FéLEZ MA, Escarrabill J*, et al.* Physical activity and its determinants in severe chronic obstructive pulmonary disease. *Med Sci Sports Exerc* 2004; **36**: 1667-1673
5. Brien SB, Lewith GT, Thomas M. Patient coping strategies in COPD across disease severity and quality of life: a qualitative study. NPJ *Prim Care Respir Med* 2016; **26:** 1-7
6. Hesselink AE, Van Der Windt D, Penninx B, *et al.* What predicts change in pulmonary function and quality of life in asthma or COPD? *J Asthma* 2006; **43:** 513-519
7. Troosters T, Blondeel A, Rodrigues FM, *et al.* Strategies to Increase Physical Activity in Chronic Respiratory Diseases. *Clin Chest Med* 2019; **40:** 397-404
8. Spruit MA, Singh SJ, Garvey C, *et al.* An official American Thoracic Society/European Respiratory Society statement: key concepts and advances in pulmonary rehabilitation. *Am J Respir Crit Care Med* 2013; **188:** e13-e64.
9. Singh D, Agusti A, Anzueto A, *et al.* Global strategy for the diagnosis, management, and prevention of chronic obstructive lung disease: the GOLD science committee report 2019. *Eur Respir J* 2019; **53:** 1900164
10. Rochester CL, Vogiatzis I, Holland AE, *et al.* An official American Thoracic Society/European Respiratory Society policy statement: enhancing implementation, use, and delivery of pulmonary rehabilitation. *Am J Respir Crit Care Med* 2015; **192:** 1373-1386.
11. Bradley JM, Moran F, Greenstone M. Physical training for bronchiectasis. *Cochrane Database Syst Rev* 2002; Issue 2: Art No CD002166
12. Global Initiative for Chronic Obstructive Lung Disease. 2021 Global Strategy for Prevention, Diagnosis and Management of COPD. Available from https://goldcopd.org/ (Accessed August 2021)
13. Bolton CE, Bevan-Smith EF, Blakey JD, *et al.* British Thoracic Society guideline on pulmonary rehabilitation in adults. *Thorax* 2013; **68 Suppl 2:** ii1-30
14. McCarthy B, Casey D, Devane D, *et al.* Pulmonary rehabilitation for chronic obstructive pulmonary disease. Cochrane Database Syst Rev 2015; Issue 2: Art No CD003793
15. Puhan MA, Gimeno-Santos E, Cates CJ, *et al.* Pulmonary rehabilitation following exacerbations of chronic obstructive pulmonary disease. Cochrane Database Syst Rev 2016; Issue 12: Art No CD005305
16. Alsubaiei ME, Cafarella PA, Frith PA, *et al.* Barriers for setting up a pulmonary rehabilitation program in the Eastern Province of Saudi Arabia. *Ann Thorac Med* 2016; **11:** 121.
17. Cox NS, Oliveira CC, Lahham A, *et al.* Pulmonary rehabilitation referral and participation are commonly influenced by environment, knowledge, and beliefs about consequences: a systematic review using the Theoretical Domains Framework. *J Physiother* 2017; **63:** 84-93.
18. Keating A, Lee A, Holland AE. What prevents people with chronic obstructive pulmonary disease from attending pulmonary rehabilitation? A systematic review. *Chron Respir Dis* 2011; **8:** 89-99.

For peer review only

19. Romain D, Bernady A, Etchamendy E, *et al.* Costs of hospitalisation for exacerbations of COPD in patients receiving domiciliary rehabilitation. *Rev Mal Respir* 2011; **28:** 864.
20. Trish E, Xu J, Joyce G. Medicare beneficiaries face growing out-of-pocket burden for specialty drugs while in catastrophic coverage phase. *Health Aff* 2016; **35:** 1564-1571.
21. Brooks D, Lacasse Y, Goldstein RS. Pulmonary rehabilitation programs in Canada: national survey. *Can Respir J* 1999; **6:** 55.
22. Wadell K, Ferreira TJ, Arne M, *et al.* Hospital-based pulmonary rehabilitation in patients with COPD in Sweden–a national survey. *Respir Med* 2013; **107:** 1195-1200.
23. Yohannes AM, Connolly MJ. Pulmonary rehabilitation programmes in the UK: a national representative survey. *Clin Rehabil* 2004; **18:** 444-449.
24. Desalu OO, Onyedum CC, Adeoti AO, *et al.* Guideline-based COPD management in a resource-limited setting - physicians' understanding, adherence and barriers: a cross-sectional survey of internal and family medicine hospital-based physicians in Nigeria. *Prim Care Respir J* 2013; **22:** 79-85.
25. Habib GM, Rabinovich R, Divgi K, *et al.* Systematic review of clinical effectiveness, components, and delivery of pulmonary rehabilitation in low-resource settings. NPJ *Prim Care Respir Med* 2020; **30:** 1-14.
26. Man WD, Puhan MA, Harrison SL, *et al.* Pulmonary rehabilitation and severe exacerbations of COPD: solution or white elephant? *ERJ Open Res* 2015; 1: 00050-2015
27. Farias CC, Resqueti V, Dias FA, *et al.* Costs and benefits of pulmonary rehabilitation in chronic obstructive pulmonary disease: a randomized controlled trial. *Braz J Phys Ther* 2014; **18:** 165-173.
28. Bove DG, Overgaard D, Lomborg K, *et al.* Efficacy of a minimal home-based psychoeducative intervention versus usual care for managing anxiety and dyspnoea in patients with severe chronic obstructive pulmonary disease: a randomised controlled trial protocol. *BMJ Open* 2015; **5:** e008031
29. Franke K-J, Domanski U, Schroeder M, *et al.* Telemonitoring of home exercise cycle training in patients with COPD. *Int J Chron Obstruct Pulmon Dis* 2016; **11:** 2821.
30. Yang F, Liu N, Hu J, *et al.* Pulmonary rehabilitation guidelines in the principle of 4S for patients infected with 2019 novel coronavirus (2019-nCoV). *Chinese Journal of Tuberculosis and Respiratory Diseases* 2020; **43:** 180-182
31. Wuytack F, Devane D, Stovold E, *et al.* Comparison of outpatient and home-based exercise training programmes for COPD: a systematic review and meta-analysis. *Respirology* 2018; **23:** 272-83
32. Chen YY, Xiao-XiaoYang FJ. Home versus centre-based pulmonary rehabilitation for patients with chronic obstructive pulmonary disease: a systematic review and meta-analysis. *TMR Integrative Medicine* 2020; **4:** 88888-e20012
33. Neves LF, Reis Md, Gonçalves TR. Home or community-based pulmonary rehabilitation for individuals with chronic obstructive pulmonary disease: a systematic review and meta-analysis. *Cad Saude Publica* 2016; **32**: e00085915
34. Dowman L, Hill CJ, Holland AE. Pulmonary rehabilitation for interstitial lung disease. *Cochrane Database Syst Rev* 2014; Issue 10: Art No CD006322
35. Taito S, Yamauchi K, Kataoka Y. Telerehabilitation in Subjects with Respiratory Disease: A Scoping Review. *Respiratory Care* 2021;66:686-698
36. Cox_NS, Dal Corso_S, Hansen_H, *et al.* Telerehabilitation for chronic respiratory disease. *Cochrane Database Syst Rev* 2021; Issue 1: Art No: CD013040

37. Higgins JPT, Thomas J, Chandler J, *et al.* Cochrane Handbook for Systematic Reviews of Interventions. Version 6.2. *Cochrane* 2021. Available from www.training.cochrane.org/handbook (Accessed August 2021)

38. Page MJ, McKenzie JE, Bossuyt PM, *et al.* The PRISMA 2020 statement: an updated guideline for reporting systematic reviews. BMJ 2021; **372:** n71

39. Casaburi R. A brief history of pulmonary rehabilitation. *Respir Care* 2008; **53:** 1185-1189

40. Cochrane Effective Practice and Organisation of Care (EPOC) group. Screening, data extraction and management. Available from https://epoc.cochrane.org/resources/epoc-resources-review-authors#conducting (Accessed August 2021)

41. Sterne JA, Hernán MA, Reeves BC, *et al.* ROBINS-I: a tool for assessing risk of bias in non-randomised studies of interventions. *BMJ* 2016; **355:** i4919

42. Higgins JP, Thompson SG, Deeks JJ, *et al.* Measuring inconsistency in meta-analyses. *BMJ* 2003; **327:** 557-560

43. Egger M, Smith GD, Schneider M, *et al.* Bias in meta-analysis detected by a simple, graphical test. BMJ 1997; **315:** 629-634

44. Schünemann H, Brożek J, Guyatt G, *et al.* GRADE handbook for grading quality of evidence and strength of recommendations. The GRADE Working Group, 2013. Available from https://gdt.gradepro.org/app/handbook/handbook.html (Accessed August 2013)

45. Holland AE, Malaguti C, Hoffman M, *et al.* Home-based or remote exercise testing in chronic respiratory disease, during the COVID-19 pandemic and beyond: a rapid review. Chronic Respir Dis 2020; **17:** 1479973120952418

For peer review only

For peer review only

Appendix 1

Search of PubMed on 12th October 2020	Search results
((((home-based rehabilitation) OR (home-based rehabilitation program) OR (home-based training) OR (home-based program) OR (home-based pulmonary rehabilitation) OR (home-based pulmonary rehabilitation program) OR (home-based exercise training) OR (home exercise) OR (home-based exercise program) OR (home-based exercise) OR (community-based rehabilitation) OR (home care services) OR (telerehabilitation) OR (tele-rehabilitation) OR (telehealth) OR (tele-health) OR (teleconsultation) OR (tele-consultation) OR (real-time videoconferencing) OR (videoconferencing) OR (telerehabilitation[MeSH Terms]) OR (home care services[MeSH Terms])))<br><br>AND ((lung disease) OR (pulmonary disease) OR (respiratory disease) OR (chronic respiratory disease) OR (chronic obstructive pulmonary disease) OR (COPD) OR (chronic obstructive airway disease) OR (chronic obstructive lung disease) OR (chronic airflow obstruction) OR (post tb) OR (post-tuberculosis) OR (interstitial lung disease) OR (idiopathic pulmonary fibrosis) OR (idiopathic interstitial pneumonia) OR (asthma) OR (occupational lung disease) OR (pulmonary hypertension) OR (lung transplant) OR (chronic bronchitis) OR (emphysema) OR (lung diseases, interstitial[MeSH Terms]) OR (bronchiectasis[MeSH Terms]) OR (idiopathic interstitial pneumonia[MeSH Terms]) OR (idiopathic pulmonary fibrosis[MeSH Terms]) OR (pulmonary disease, chronic obstructive[MeSH Terms]) OR (lung transplantation[MeSH Terms])))<br><br>AND ((pulmonary rehabilitation) OR (cardiopulmonary rehabilitation) OR (respiratory therapy) OR (respiratory muscle training) OR (breathing exercise) OR (pulmonary exercise) OR (pre-habilitation) OR (breathing exercises[MeSH Terms]) OR (respiratory therapy[MeSH Terms])) AND (1990:2020[pdat])	4058

For peer review only

Reporting checklist for protocol of a systematic review and meta analysis.

Based on the PRISMA-P guidelines.

Instructions to authors

Complete this checklist by entering the page numbers from your manuscript where readers will find each of the items listed below.

Your article may not currently address all the items on the checklist. Please modify your text to include the missing information. If you are certain that an item does not apply, please write "n/a" and provide a short explanation.

Upload your completed checklist as an extra file when you submit to a journal.

In your methods section, say that you used the PRISMA-Preporting guidelines, and cite them as:

Moher D, Shamseer L, Clarke M, Ghersi D, Liberati A, Petticrew M, Shekelle P, Stewart LA. Preferred Reporting Items for Systematic Review and Meta-Analysis Protocols (PRISMA-P) 2015 statement. Syst Rev. 2015;4(1):1.

		Reporting Item	Page Number
**Title**			
Identification	#1a	Identify the report as a protocol of a systematic review	2
Update	#1b	If the protocol is for an update of a previous systematic review, identify as such	n/a
**Registration**			
	#2	If registered, provide the name of the registry (such as PROSPERO) and registration number	PROSPERO CRD42020220137
**Authors**			

For peer review only

Contact	#3a	Provide name, institutional affiliation, e-mail address of all protocol authors; provide physical mailing address of corresponding author	1
Contribution	#3b	Describe contributions of protocol authors and identify the guarantor of the review	8
**Amendments**			
	#4	If the protocol represents an amendment of a previously completed or published protocol, identify as such and list changes; otherwise, state plan for documenting important protocol amendments	n/a
**Support**			
Sources	#5a	Indicate sources of financial or other support for the review	8
Sponsor	#5b	Provide name for the review funder and / or sponsor	8
Role of sponsor or funder	#5c	Describe roles of funder(s), sponsor(s), and / or institution(s), if any, in developing the protocol	8
**Introduction**			
Rationale	#6	Describe the rationale for the review in the context of what is already known	4
Objectives	#7	Provide an explicit statement of the question(s) the review will address with reference to participants, interventions, comparators, and outcomes (PICO)	5,11,12
**Methods**			
Eligibility criteria	#8	Specify the study characteristics (such as PICO, study design, setting, time frame) and report characteristics (such as years considered, language, publication status) to be used as criteria for eligibility for the review	11, 12
Information sources	#9	Describe all intended information sources (such as electronic databases, contact with study authors, trial registers or other grey literature sources) with planned dates of coverage	5

For peer review only

Search strategy	#10	Present draft of search strategy to be used for at least one electronic database, including planned limits, such that it could be repeated	5
Study records - data management	#11a	Describe the mechanism(s) that will be used to manage records and data throughout the review	
Study records - selection process	#11b	State the process that will be used for selecting studies (such as two independent reviewers) through each phase of the review (that is, screening, eligibility and inclusion in meta-analysis)	6
Study records - data collection process	#11c	Describe planned method of extracting data from reports (such as piloting forms, done independently, in duplicate), any processes for obtaining and confirming data from investigators	6
Data items	#12	List and define all variables for which data will be sought (such as PICO items, funding sources), any pre-planned data assumptions and simplifications	6, 11, 12
Outcomes and prioritization	#13	List and define all outcomes for which data will be sought, including prioritization of main and additional outcomes, with rationale	6
Risk of bias in individual studies	#14	Describe anticipated methods for assessing risk of bias of individual studies, including whether this will be done at the outcome or study level, or both; state how this information will be used in data synthesis	6
Data synthesis	#15a	Describe criteria under which study data will be quantitatively synthesised	7
Data synthesis	#15b	If data are appropriate for quantitative synthesis, describe planned summary measures, methods of handling data and methods of combining data from studies, including any planned exploration of consistency (such as I2, Kendall's τ)	7
Data synthesis	#15c	Describe any proposed additional analyses (such as sensitivity or subgroup analyses, meta-regression)	7
Data synthesis	#15d	If quantitative synthesis is not appropriate, describe the type of summary planned	7

For peer review only

Meta-bias(es)	#16	Specify any planned assessment of meta-bias(es) (such as publication bias across studies, selective reporting within studies)	6
Confidence in cumulative evidence	#17	Describe how the strength of the body of evidence will be assessed (such as GRADE)	7

None The PRISMA-P elaboration and explanation paper is distributed under the terms of the Creative Commons Attribution License CC-BY. This checklist can be completed online using https://www.goodreports.org/, a tool made by the EQUATOR Network in collaboration with Penelope.ai

For peer review only
